# Long-Term Recency in Anterograde Amnesia

**DOI:** 10.1371/journal.pone.0124084

**Published:** 2015-06-05

**Authors:** Deborah Talmi, Jeremy B. Caplan, Brian Richards, Morris Moscovitch

**Affiliations:** 1 Department of Psychology, University of Toronto, Toronto, Ontario, Canada; 2 School of Psychological Sciences, University of Manchester, Manchester, United Kingdom; 3 Rotman Research Institute, Baycrest, Toronto, Ontario, Canada; 4 Psychology Department and Centre for Neuroscience, University of Alberta, Edmonton, Alberta, Canada; 5 Department of Psychology, Baycrest Centre, Toronto, Ontario, Canada; Alexander Fleming Biomedical Sciences Research Center, GREECE

## Abstract

Amnesia is usually described as an impairment of a long-term memory (LTM) despite an intact short-term memory (STM). The intact recency effect in amnesia had supported this view. Although dual-store models of memory have been challenged by single-store models based on interference theory, this had relatively little influence on our understanding and treatment of amnesia, perhaps because the debate has centred on experiments in the neurologically intact population. Here we tested a key prediction of single-store models for free recall in amnesia: that people with amnesia will exhibit a memory advantage for the most recent items even when all items are stored in and retrieved from LTM, an effect called long-term recency. People with amnesia and matched controls studied, and then free-recalled, word lists with a distractor task following each word, including the last (continual distractor task, CDFR). This condition was compared to an Immediate Free Recall (IFR, no distractors) and a Delayed Free Recall (DFR, end-of-list distractor only) condition. People with amnesia demonstrated the full long-term recency pattern: the recency effect was attenuated in DFR and returned in CDFR. The advantage of recency over midlist items in CDFR was comparable to that of controls, confirming a key prediction of single-store models. Memory deficits appeared only after the first word recalled in each list, suggesting the impairment in amnesia may emerge only as the participant’s recall sequence develops, perhaps due to increased susceptibility to output interference. Our findings suggest that interference mechanisms are preserved in amnesia despite the overall impairment to LTM, and challenge strict dual-store models of memory and their dominance in explaining amnesia. We discuss the implication of our findings for rehabilitation.

## Introduction

Anterograde amnesia is defined as an impairment of memory retrieval from a long-term memory (LTM) store accompanied by intact retrieval from a short-term memory (STM) store or working memory [1–3], a definition expressed in many modern textbooks [4, 5]. This view of amnesia assumes a dual-store model of memory [6], and has persisted despite an ongoing debate between dual-store and single-store models on explaining memory behaviour in healthy participants [7–13] and a variety of findings from neuropsychology, neuroimaging and animal models of memory [14]. Provocatively, even Crowder, who argues against dual-store models, pointed out that his own textbook expressed the dual-store view [15, 16]. Here we focus on single-store models and a key prediction they make for amnesia: that people with amnesia would exhibit an intact long-term recency effect. As we discuss below, this prediction also has important value for rehabilitation. Before we turn to this specific prediction, however, we first provide some background for this debate to clarify why this prediction is important.

### Dual store models

The clearest and most direct support of early, “classical” dual-store models of amnesia was the finding that healthy participants recall late list items better than midlist and early items in immediate free recall (IFR) but not in delayed free recall (DFR) [17]. IFR tasks typically present participants with a list of words and they are immediately asked to recall as many words as they can, regardless of the order of presentation. The serial position curve is a plot of the number of words recalled as a function of the position of each word in the study list, and in IFR it has a U-shape due to the presence of both recency and primacy effects. In DFR, a distracting task is added at the end of the list, prior to the recall test. The presence of recency in IFR was compatible with the notion that final list items are retrieved easily from STM whereas earlier list items are retrieved from LTM, a process more prone to failure [18–20]. To explain the near-flatness of the serial position curve in DFR classical dual-store models proposed that the distractor task in DFR displaces final list items from STM so that at test they, too, need to be retrieved from LTM, and that retrieval from LTM does not in any way favour the most recently presented words. Support for classical dual-store models dwindled when a recency effect was observed in a procedure known as continual-distractor free recall (CDFR), where a distracting task is interpolated after each word in the study list [1, 20, 21]. The recency effect in CDFR was termed “long-term recency” to make the point that if dual-store models were correct, this recency effect must have been due to retrieval from LTM. Because the end-of-list distractor is common to both DFR and CDFR, classical dual-store models predicted a flat, recency-less serial position curve in CDFR. The presence of long-term recency was therefore held as evidence against these models [1, 16, 22–26] and this has been confirmed in dual-store model simulations [27].

### Single store models

Single-store models assume that in free recall, all studied items compete to be retrieved. Competition is related to the similarity between the retrieval context and the study context, which partially depends on how much more recent an item is compared to all other studied items. For example, assume a presentation rate of one word per second and end-of-list distractor lasting 10 seconds. In IFR, at the start of recall, the last item was presented 1 s ago, but the second-last item was presented 2 s ago. The second-last item is thus twice as far in the past as the last item, so the last item will compete well against the second-last and previous list items. In DFR, the recency of the last list-item is 11 s (1 distractor’s plus 1 item's worth of time), and the recency of the second-last item is 12 s. The last two items are nearly equal in recency, which means the competitive advantage of the last item over earlier list items is greatly reduced. In CDFR, the recency of the last item is again 11 s, but the recency of the second-last item is 22 s (2 distractors' plus 2 items' worth of time). The last item thus once again has a large competitive advantage (here, two-fold) over earlier list items. Because recall is assumed to be competitive, the ratio of recencies between a pair of items determines how likely one item is to be recalled versus another [21]. The assumption that probability of recall depends on the pattern of *relative* recencies and not absolute time, an assumption known as the *ratio rule*, makes interference-theory-based models scale-invariant [16]. Unlike classical dual-store models, long-term recency is a natural prediction of interference-theory models of memory, which only posited a single memory store. Therefore, the long-term recency effect was a cornerstone in their ascendance over early dual-store models [7–12].

Single store models could also predict the pattern of deficits in amnesia [11, 28, 29], and claim, in the interest of parsimony, that the debate is settled in favour of the single store model. Because this claim rests primarily on evidence of long-term recency effects in healthy controls, and on only one study in amnesia [22], the dominant view of amnesia is still firmly grounded in the classical dual store model.

### Hybrid models

Baddeley and Hitch [13] claim that interference-based models of memory fare poorly in explaining very long term recency, for instance, when recalling the results of a sport season a long while after it is over (Baddeley & Hitch, 1977). In such situations the retrieval context is a lot more different from the context of encoding of multiple game instances, and it is less likely that similarity would favour memory for the most recent game. To resolve this they propose an alternative model of recency, called the Activation Model. In this model recently presented items are more strongly ‘activated’ than other items. In indirect tests of memory this activation would yield priming effect. Recency effects in direct tests of long-term memory tests ensue when participants adopt a strategy of retrieving more highly activated items first. The differences between this model of long-term memory and other interference-based models still need to be worked out. The Activation Model could be considered a ‘hybrid’ model of memory because the authors assume that this process could occur in multiple memory stores, and do not commit to a single store view [13]. A model that is ‘hybrid’ by design was suggested by Davelaar et al (2005). Their model assumes two memory stores, STM and LTM, and accommodates the long-term recency effect by assuming that LTM functions as interference-based models predict.

The performance of people with amnesia on IFR has been researched extensively, and accords with the predictions of both dual and single store models. People with amnesia exhibit an intact recency effect in IFR accompanied by a reduced probability of recalling items from earlier in the list [30–35]. This pattern is compatible with the notion that final list items are retrieved from an intact short-term memory store, whereas earlier items are retrieved from an impaired LTM. It is also compatible with interference-based single-store models and the activation model or recency, which assume that recent items have a retrieval advantage. To account for poorer retrieval of earlier items in amnesia, these views could refer to reduced rehearsal [36] or increased susceptibility to interference [37–41]. The performance of people with amnesia on CDFR has not been established.

Crucially, given the scale-invariance that interference-based single store models of memory assume, they must predict that whatever memory is spared in amnesia must also be scale-invariant; hence, these models predict an intact long-term recency effect in amnesia, equivalent in magnitude to the effect in controls. Classical dual-store models cannot account for long-term recency in any population. Finally, hybrid models do not need to predict scale invariance because they assume that memory operates differently at different time scales.

Taken together, a demonstration of long-term recency in amnesia is of theoretical interest because it would only provide a strong support for single-store models. Both single-store and hybrid models can account for the full long-term recency pattern, namely recency in IFR which is reduced in DFR and then returns in CDFR (hybrid models do so at the cost of reduced parsimony); but only single-store models predict that the LTR should be unaltered in amnesia (equivalent to controls).

The question of whether or not people with amnesia exhibit a long-term recency effect is not only of theoretical interest. Currently, in accordance with the classical, early dual store model definition of amnesia, patients are thought not to be able to retain any information in LTM. Although clinical observations show that patients do sporadically report such information, and free-recall intrusions from previous list have also been documented in the research literature [13, 42], these reports are not thought to be governed by any known rules and are, therefore, given little weight.

To emphasize the clinical relevance of our findings we attempted to demonstrate the ecological validity of the long-term recency effect in this group. For this purpose we designed an incidental song-recall task. Participants listened to one song after each free-recall period (following each of the study lists) and were asked to recall the songs freely at the end of the testing session. The song-recall task can be viewed as a form of (incidental) continual-distractor free recall procedure, in which the word-list task takes on the role of the distractor. If participants with amnesia exhibit long-term recency in CDFR, they should also recall more of the songs they studied late in the course of the experiment relative to those they studied at the beginning of the session.

Our work follows up on a previous attempt to characterise long-term recency in amnesia. Carlesimo and colleagues [22] first tested for long-term recency in people with amnesia. They described their results as people with amnesia exhibiting an intact recency effect in IFR, in line with previous work [30–35], but an impaired recency effect in CDFR compared to neurologically intact controls. This would be an important finding because it cannot be accommodated by single-store models of memory, which predict scale invariance. However, the authors’ interpretation is not the only possible view of their results. What they actually found in the CDFR condition was an equivalent-sized reduction in probability of recall at all serial positions. Equality of recency slopes is the relevant prediction of interference-based single-store models and the activation model of recency, and visual inspection of the data suggests that the slopes of the recency effects were quite similar for patients as for controls in their CDFR condition. More seriously, as also noted by others [11], the authors used an entirely different methodology in the IFR and CDFR conditions; in the IFR condition, Carlesimo et al.’s participants were given a list of words to study and recall, whereas in CDFR, participants were given the list of words as anagrams to solve, with the unscrambled words being the target material for subsequent recall (a paradigm previously used by Vallar, Papagno and Baddeley, 1991). This difference in methodologies between the IFR and CDFR conditions makes it difficult to draw inferences across the two tasks. For example, although anagram solution generally motivates incidental study, controls may have rehearsed more than patients [7] during the distractor tasks, which could have aided their retrieval of the final list items and artificially increased their long-term recency. It could be that in the CDFR task, there was simply a group difference (main effect) in overall difficulty of the task, and if they had used the same anagram task during their IFR condition, perhaps the main effect would have been evident in that task as well.

Moreover, Carlesimo et al.’s study is made even more inconclusive because it lacked the DFR condition, which is an essential component of the traditional long-term recency debate. It is the flattening of the recency effect in the DFR condition that establishes that the distractor task is effective (either in emptying STM, from the perspective of dual-store models, or in providing a sufficiently strong contextual shift, from the perspective of interference-theory based models). Without the DFR condition, we do not know if the distractor task was equally effective across amnesic and control groups. Moreover, we don’t even know if *either* group exhibited long-term recency by the standard definition, which demands: a) recency in the IFR condition, plus b) a flattening of the recency effect in the DFR condition, followed by c) a return of recency in the CDFR condition, exceeding that in the DFR condition. In other words, the basic validity tests were missing. A flattened recency effect in DFR that then returned in CDFR would militate against the criticism above. Here we applied the full set of conditions (IFR, DFR and CDFR) to both people with amnesia and neurologically intact controls, using the same procedure across conditions and groups. Finally, because amnesic participants are often found to be at a disadvantage in intentional memory tasks but unimpaired in incidental and implicit memory tasks (e.g. [43]), we employed an intentional study test to provide a stronger test of long-term recency in amnesia.

## Material and Methods

### Participants

13 patients (two females) and 15 healthy control subjects (two females) participated in this study. Patients were referred to the study by their consultant, who ensured that the patients had the ability to consent for themselves. Patient's consent was always obtained. No next of kin were involved in the patients' contribution to the research project. The study received the approval of the Toronto Academic Health Sciences Network ethics review board, who reviewed and confirmed the consent procedure.

Patients and controls were matched on age (t<1), years of education (t<1), digit span forward (t<1) or backward [t(24) = 1.21, p>.10] and premorbid intelligence as assessed by the Wechsler Adult Test of Reading (WTAR, t<1), see Table 1. 12 of the patients were referred to the Memory Link program in Baycrest, Toronto, Canada (http://research.baycrest.org/mlp) because of impaired memory caused by a variety of traumatic disorders such as ruptured aneurysm and encephalitis. Patient KC was originally referred to MM in 1983 following a closed head injury [44] and has volunteered in our studies since then. The performance of these patients on a number of key neuropsychological tests was retrieved from their current hospital file. To minimize the effect of frontal-lobe damage on our amnesic sample, we excluded data from three patients who were impaired on both phonemic fluency and the Wisconsin Card Sorting Test. An additional patient was excluded from all further analysis because she recalled more midlist than recency words in IFR, unlike any of the other patients or controls. This decision was taken on the basis that it is difficult to interpret long-term recency in a participant who does not exhibit recency in IFR. As shown in Table 2, all the 9 patients included in our final sample had severely impaired performance on tests of long term memory as assessed by CVLT and BVT, accompanied by preserved digit span, intelligence and normal function on at least one of the tests sensitive to frontal damage. Thus, although the etiologies leading to amnesia differ among the patients, the functional consequences are the same with respect to loss of LTM but relatively preserved STM as these functions are defined on standard tests. This functional impairment is the starting point, and focus, of our study.

**Table 1:** Characteristics of patient and control participants.

	Controls		Patients	
	Mean	SD	Mean	SD
Age	55.33	7.06	52.56	5.13
Years of Education	16.00	2.36	16.22	3.15
Premorbid intelligence	110.47	10.07	109.89	11.23
Digits span forward	7.27	1.33	7.11	0.93
Digits span backward	5.53	1.46	5.00	1.66

*Note*. SD = standard deviation. Premorbid intelligence was assessed using the Wechsler Test of Adult Reading.

**Table 2:** Neuropsychological characteristics for participants with Amnesia.

	PR	MS	JSB	DA	MT	RR	KM	MB	KC
**Male/Female**	M	M	M	M	M	F	F	M	M
**Age**	47	52	59	55	42	**56**	**53**	**55**	54
**Aetiology**	Seizures	Encephalitis	Korsakoff	Encephalitis	Aneurism	Cyst removal	Aneurism	Aneurism	Head Trauma
**WTAR**	106	120	119	117	92	96	119	119	101
**WASI**									
FSQ	82%ile	87%ile	92%ile	87%ile	High average*	81%ile	55%ile	90%ile	47%ile
VSQ	86%ile	94%ile	96%ile	92%ile	Average*	81%ile	12%ile	91%ile	47%ile
PSQ	70%ile	47%ile	77%ile	66%ile	Superior*	73%ile	96%ile	86%ile	47%ile
**Digit Span**									
Total	10	18	25	18	16	22	16	21	17
Forward	5	8	8	7	7	7	7	7	8
Back	3	5	7	5	4	7	4	7	3
**CVLT**									
Immediate recall	4%ile	25%ile	2%ile	<1%ile	<1%ile	4%ile	<1%ile	3%ile	<1%ile
Short delay	0	1	0	0	0	0	2	4	<1%ile
Long delay	0	1	1	0	0	0	0	2	<1%ile
**BVMT**									
Immediate recall	0%ile**	<1%ile	2%ile	1%ile	<1%ile	<1%ile	7%ile	♯	7%ile
Delayed recall	0%ile**	1%ile	4%ile	<1%ile	<1%ile	<1%ile	7%ile	1%ile♯	<1%ile
Recognition	<1%ile**	11–16%ile	6–10%ile	<1%ile	11–16%ile	1–2%ile	>16%ile	♯	1–2%ile
**Fluency**									
Phonemic (FAS)	≠	45%ile	63%ile	98%ile	30%ile	16%ile	16%ile	<1%ile	7–13%ile
Semantic (animals)	≠	19%ile	37%ile	50%ile	10%ile	12%ile	99%ile	<1%ile	50%ile
**WCST**	>16%ile	>16%ile	>16%ile	>16%ile	>16%ile	>16%ile	_≠	>16%ile	>16%ile

*Notes*. WASI = Wechsler Abbreviated Test of Intelligence; FSQ = full scale quotient; VSQ = visual scale quotient; PSQ = performance scale quotient; WTAR = Wechsler Test of Adult Reading; CVLT = California Verbal Learning Test; BVMT = Brief Visual Memory Test; FAS = Letter fluency test; WCST = Wisconsin Card Sorting Test. The values given are the raw scores when they were available; otherwise we noted the percentile scores.

*This description was the only "score" provided for us.

**The complex figure of the Kaplan Baycrest Neuropsychological Assessment (KBNA) was administered instead of the BVMT.

^≠^Not tested in this patient.

^♯^The Rey-Osterrieth Complex Figure Test was administered instead of the BVMT.

Control participants were recruited from the University of Toronto and the Rotman Research Institute volunteer pool. The study received the approval of the Toronto Academic Health Sciences Network ethics review board.

### Materials

#### Word stimuli

Nine word lists were constructed, and three lists were used in each one of three experimental tasks: IFR, DFR, and Each word lists consisted of 9 words. Six of the words in each list were randomly sampled without replacement for each participant from the same pool of 78 words. In addition, to these six words, three ‘target’ words in each list were sampled without replacement from a separate pool of 27 words and placed randomly in serial positions 1, 5, and 9. The 3-letter stem of target words had between 4 and 9 accessible completions (M = 5.78, SD = 1.7%) and the average probability of each word to be given as a first response to the stem ranged from 2% to 9.3% (M = 4.1%, SD = 2.2% according to published norms [45]). None of the target words had the same stem as another word in the experiment. All experimental words were nouns, had 5–6 letters, mean frequency of 41.62, SD = 54.68 [46], mean concreteness of 553, SD = 67.37 [47], and all were neutral in valence. To reduce proactive interference, words for practice trials were all proper names, sampled from a pool of 30 male and female first names. Words were presented in lowercase, black 72-point Times New Roman font on a yellow background.

#### Distractor task stimuli

The stimuli for the distractor task were simple yet cognitively demanding arithmetic problems. Each included three randomly sampled single digits and a solution which could be correct or incorrect. Two of the digits had values between 1–5 and one had values between 1–9. Incorrect solutions were larger or smaller than the correct one by 1 (for example, 9+4+2 = 16 or 3+8+1 = 12). The problems were presented on a grey background in the center of to the left or the right of the center of a grey screen, in black 28-point Times New Roman font.

#### Song excerpts

All songs used in the song-recall task were highly familiar, uplifting, popular American songs, such as ‘Rock around the Clock’ or ‘Singing in the Rain.’ To increase the chances that most participants, even those with retrograde amnesia, would be familiar with most of the songs, we selected songs which were mostly released in the fifties and sixties; the most recent song used was ‘American Pie,’ released in 1971. One-minute excerpts from each song, which included the most familiar part (e.g. the chorus), were converted to 22050 samples, 8-bit stereo files. One song was played after each list for a total of nine songs in all. Song order was randomized for each participant.

### Procedure

**Fig 1:**
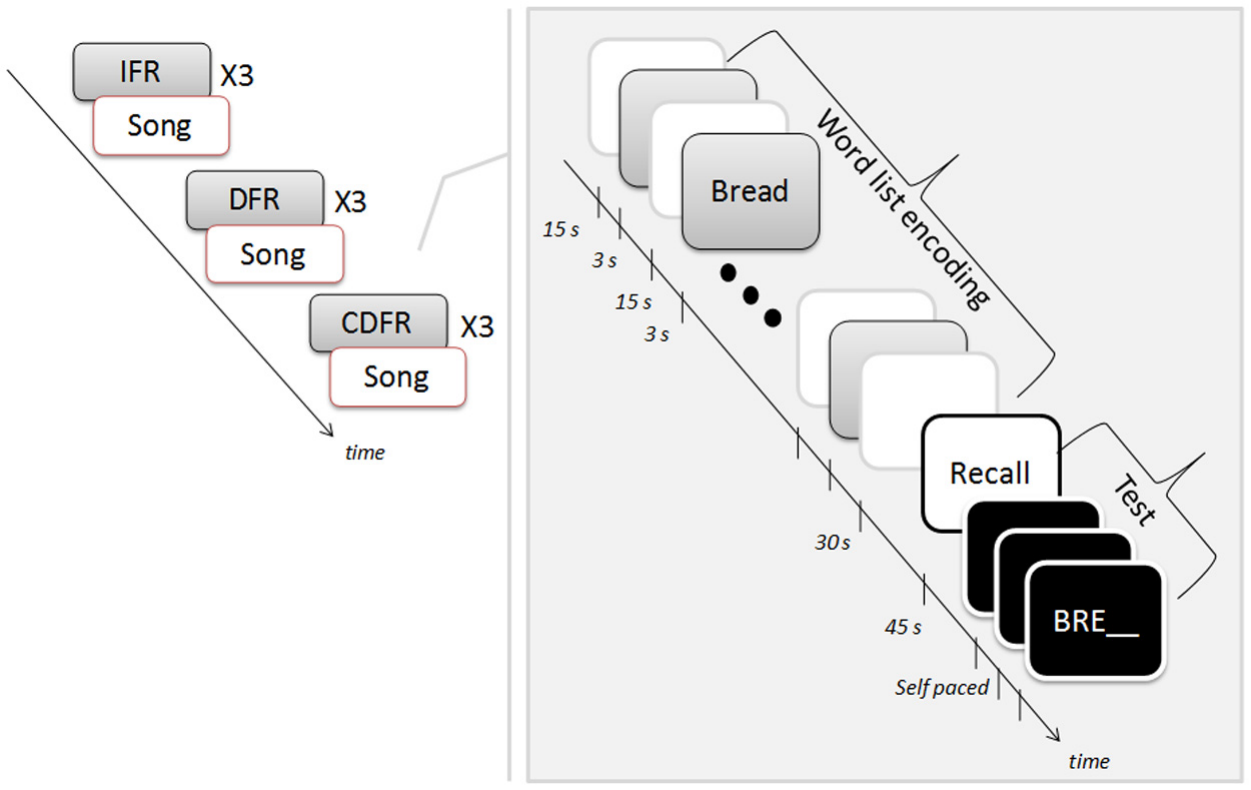
Task structure. Participants saw 9 word lists, 3 in each of 3 conditions—IFR, DFR, and CDFR. After each list a song was played for 1 minute, and participants’ familiarity with the song was assessed. The CDFR condition is illustrated in more detail on the right with timing information shown under the timeline arrow. This condition included the presentation of three 9-word lists (grey screens). An inter-word interval preceded each of the 9 words (white screens with grey frames) and was filled with an arithmetic task. A longer interval was inserted after the last word, just before the recall test. At the end of that final interval participants saw the word ‘Recall’ and invited to freely recall the words they remembered (white screen with black frame). They were then given a cued recall for three of the words (black screens).

Prior to the experiment itself, participants received initial practice which included studying and recalling three 4-word lists, and performing the distractor task three times. Following initial practice, all participants were able to answer at least one distractor question and recall at least one word. The order of the three experimental tasks was counterbalanced across participants. Each task began with instructions and one practice list, and included three word lists. Fig 1 depicts the encoding and testing procedure for each list. Participants were reminded of the instructions before each list. The experimenter recorded all words participants recalled. A five-minute break followed each task. During the first break, participants completed the WTAR. There was no task during the second break. The digit span task (forward and backward) was administered after the last task. Finally participants were reminded that some songs were played during the course of the experiment and were invited to take as much time as they needed to recall freely the names of these songs.

#### Immediate free recall (IFR)

Words were presented for 3 seconds each. The orienting task for encoding required participants to rate each word as either ‘pleasant’ or ‘unpleasant’. They rated the words by pressing one of two marked keys on a computer keyboard; the rest of the keyboard was covered with cardboard in order to make it easier for patients to switch back to the orienting task in CDFR. A blank inter-stimulus interval (ISI) of 500 ms followed each word. The instruction ‘Recall words’ was presented after the final ISI and participants were given 45 seconds to recall studied words in any order.

#### Delayed free recall (DFR)

The procedure was almost identical to IFR, except that following the last ISI participants performed the distractor task for 30 seconds.

#### Continual-distractor free recall (CDFR)

The procedure was almost identical to that of the IFR task, but instead of the blank ISI which followed each word, participants performed the distractor task for 15 seconds. They also performed the distractor task for 15 seconds once before the first word, and for 30 seconds after the last studied word.

#### Distractor task

The computer monitor changed colour to grey when the task began. A single arithmetic problem was presented, and participants read it out loud, and say ‘yes’ if the solution was correct, and ‘no’ if it was incorrect. The experimenter keyed in their response, which triggered the next problem.

#### Cued recall

This test provides more support during retrieval than free recall and was included to allow even the most densely amnesic participants to experience some success in retrieving studied words so as to motivate them during the course of this challenging experiment. The instructions encouraged participants to first think back about the studied list and attempt to retrieve a studied word, but if they could recall no appropriate completion, to complete the stem with the first word that came to mind [48]. The cued recall test began as soon as participants completed each free-recall test and consisted of a presentation of the stems for each one of the three target words (serial positions 1, 5 and 9) in a random order. Participants verbally completed each presented stem before the next stem appeared. Results from this test are reported in Table 3 but because of potentially differential reliance on implicit memory in the patient and control groups they are not analysed further.

**Table 3:** Cued recall performance.

	IFR1	IFR2	IFR3	CDFR1	CDFR5	CDFR9	DFR1	DFR5	DDFR9
patients	0.63 (0.35)	0.56 (0.24)	0.63 (0.39)	0.33 (0.37)	0.33 (0.37)	0.30 (0.35)	0.52 (0.38)	0.41 (0.36)	0.44 (0.24)
controls	0.87 (0.21)	0.73 (0.29)	0.82 (0.28)	0.49 (0.42)	0.60 (0.31)	0.60 (0.34)	0.76 (0.29)	0.62 (0.33)	0.62 (0.38)

Note. The average number (and SD) of words participants provided in a cued recall test after IFR, DFR and CDFR tests. The numbers (1, 5, and 9) refer to the serial positions of the studied words to which the cues referred. For example, patients given a cue for the first word they studied in IFR retrieved, on average, 0.63 of a word, compared to controls, who retrieved 0.87 of a word.

#### Song encoding

Immediately following each cued recall test, participants were instructed to relax and listen to a single song excerpt. They then answered the following questions about it: (1) Is the song familiar to you? (2) What is the name of the song? (3) Do you know all, some, or none of the words? (4) Can you hum all, some, or none of the tune? All participants enjoyed the song task and experienced success in it, which helped motivate those who found recalling the words particularly challenging.

#### Adapting the recall task to people with amnesia

Task switching between word study and the distractor task was more difficult for patients than controls, because they did not always remember the complete set of instructions. When patients failed to rate a word, or did not immediately begin reading the arithmetic problems, the experimenter gave short reminders, either pointing at the keyboard or at the screen, or providing short verbal cues (‘read the exercise’, ‘rate the word’). Following the practice list, patients were able to perform the tasks adequately with minimum reminders. The necessity of these reminders for patients but not controls was the only difference in procedure between the two groups.

## Results

All data is freely available to download from the first author’s university article repository (https://www.escholar.manchester.ac.uk).

### Free recall of words

Free recall was computed as the number of matches between words presented and words produced across the three free-recall tests in each condition, separately for each serial position. We used the Greenhouse-Geisser correction for violations of sphericity whenever necessary and analysed polynomial trends to the fourth order.

**Fig 2:**
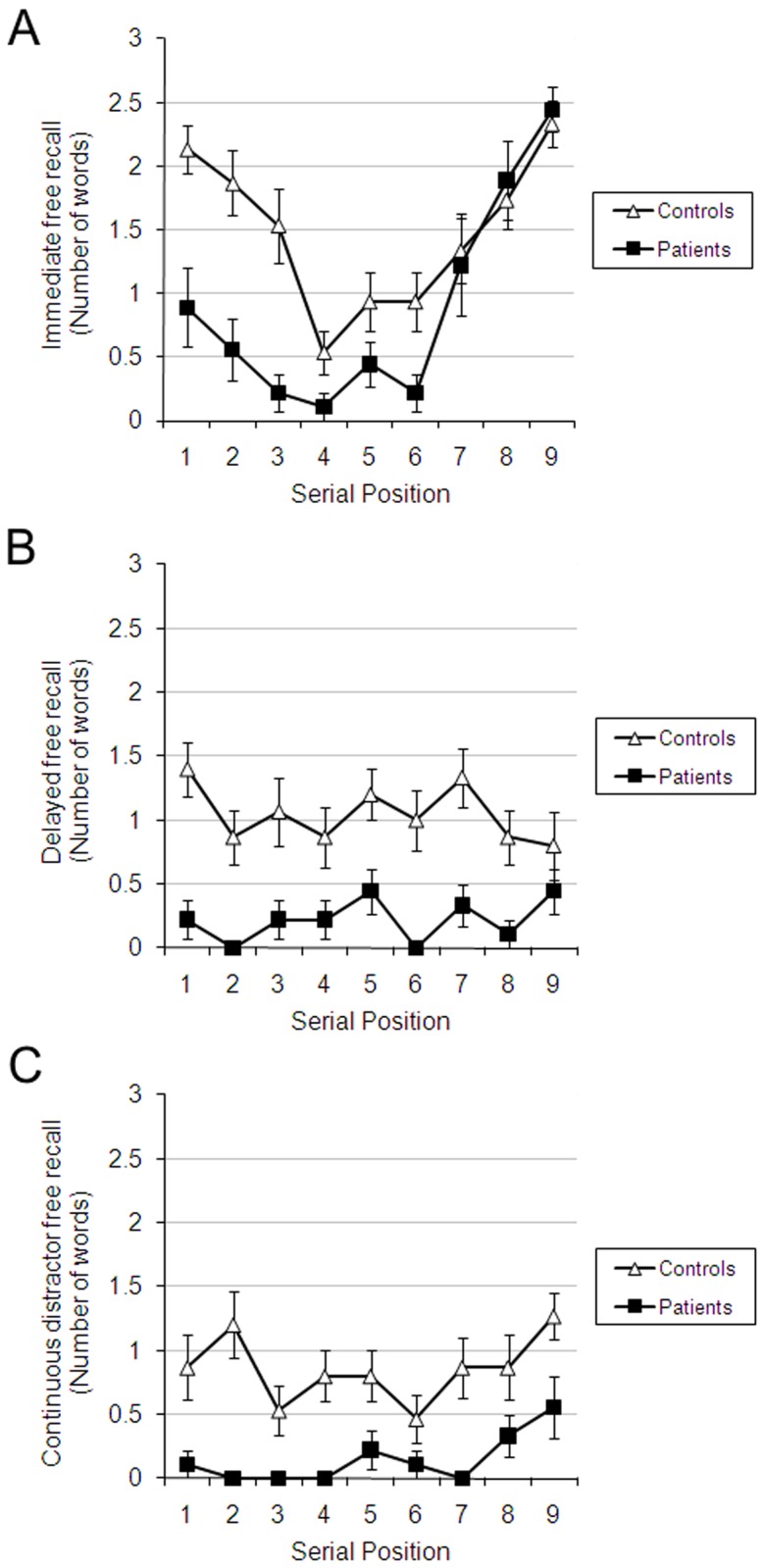
Number of words recalled as a function of group and serial position. (a) IFR (b) DFR (c) CDFR. Error bars represent standard error.

**Fig 3:**
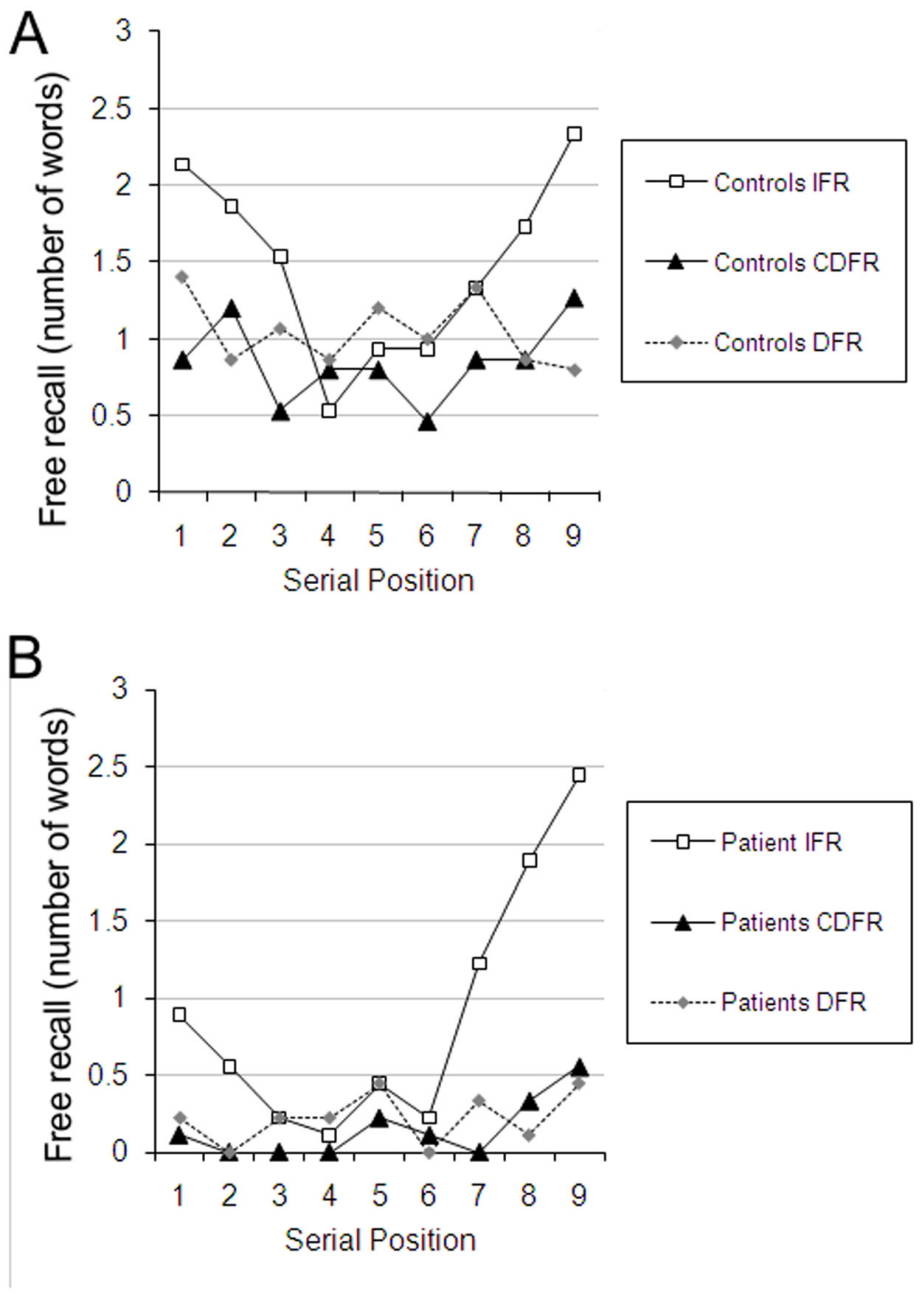
Number of words recalled as a function of group and serial position. Here the data are re-plotted from Fig 2 with all conditions (IFR, DFR and CDFR) plotted for Controls (a) and Amnesia (b) separately. Note that both groups show a reduction of recency in DFR compared to IFR, and a return of recency in CDFR compared to DFR.

Fig 2 plots the data as in Carlesimo et al. (1996). This figure shows that both patients and controls exhibited a recency effect in IFR (Fig 2a) and CDFR (Fig 2c), but not in DFR (Fig 2b), the condition that was absent in Carlesimo et al.’s study, confirming that the distractor task was effective for both groups. Although patients exhibited a recency effect (greater recall of the end-of-list items than earlier items) in both IFR and CDFR, the number of words they recalled of the late portion of the study list equalled that of controls only in IFR, not in CDFR. In CDFR, patients’ memory (probability of recall) was depressed for all serial positions relative to controls, replicating Carlesimo et al. (1996). Fig 3 plots the data in a way that makes it easier to compare the conditions within each group, and shows that the long-term recency effect was present within each group in its own right.

Free-recall data were analysed with a Task (IFR, DFR, CDFR) x Serial position (1–9) repeated-measures ANOVAs with Group (patients, controls) as a between-subjects factor. The main effects of Task [F(2,44) = 45.02, p<.001, partial η^2^ = .67] and Serial Position [F(8, 176) = 9.07, p<.001, partial η^2^ = .29] were significant, as well as the interaction between them [F(16,352) = 5.98, p<.001, partial η^2^ = .21]. The main effect of Group [F(1,22) = 30.04, p<.001, partial η^2^ = .58] was significant, and it interacted with Serial Position [F(8,176) = 2.24, p<.05, partial η^2^ = .09]. The three-way interaction did not reach significance [F(16,352) = 1.38, p>.10, partial η^2^ = .06]. To increase the power of this analysis, and following Carlesimo et al. (1996), we averaged serial positions 7-8-9 (recency), 4-5-6 (midlist), and 1-2-3 (primacy) and ran the analysis again. The same pattern was obtained, but this time the 3-way interaction was significant [F(4,88) = 3.14, p<.05, partial η^2^ = .12].

We explored the 2-way interaction between Task and Serial Position by analysing each task separately, using Serial Position as a within-subject factor and Group as a between-subject factor. In IFR there was a significant effect of Serial Position [F(8,176) = 15.86, p<.001, partial η^2^ = .42], which exhibited a linear [F(1,22) = 11.60, p<.01, partial η^2^ = .34] and a more pronounced quadratic [F(1,22) = 98.20, p<.001, partial η^2^ = .82] trend, consistent with the presence of primacy and recency effects. The effect of Group was significant [F(1,22) = 13.07, p<.01, partial η^2^ = .37] and Group interacted with Serial Position [F(8,176) = 3.25, p<.01, partial η^2^ = .13]. To explore this interaction we carried out a series of Bonferroni-corrected one-tailed t-tests on recall from primacy, midlist and recency positions, computed as described above. These revealed that this interaction was due to the fact that relative to patients, controls recalled more primacy [t(22) = 4.35, p<.005] and midlist [t(22) = 2.69, p<.01] items, but there was no significant difference between the groups in recall of recency items (t<1). As expected, both controls [t(14) = 5.12 p<005] and patients [t(8) = 6.61, p<.005] recalled more recency than midlist items. In CDFR, Serial Position had a significant effect on probability of recall [F(8,176) = 1.99, p = .05, partial η^2^ = .08], an effect which exhibited a quadratic trend [F(1,22) = 6.50, p<.05, partial η^2^ = .23]. The main effect of Group was significant [F(1,22) = 20.23, p<.001, partial η^2^ = .48] but Group did not interact with Serial Position [F<1]. Recency items were recalled more often than midlist items when both groups were considered together [t(23) = 2.53, p<01, one-tailed]. Although the interaction with Group was not significant, for completion we report that one-tailed t-tests confirmed that this effect remained significant when each group was examined separately [controls: t(14) = 1.93, p<.05; patients: t(8) = 2.29, p<.05]. Serial Position did not have a significant effect on DFR, [F(8,176) = 1.07, p>.10, partial η^2^ = .05]. The main effect of Group was significant [F(1,22) = 30.86, p<.001, partial η^2^ = .58] but Group did not interact significantly with Serial Position [F<1].

### Probability of first recall

**Fig 4:**
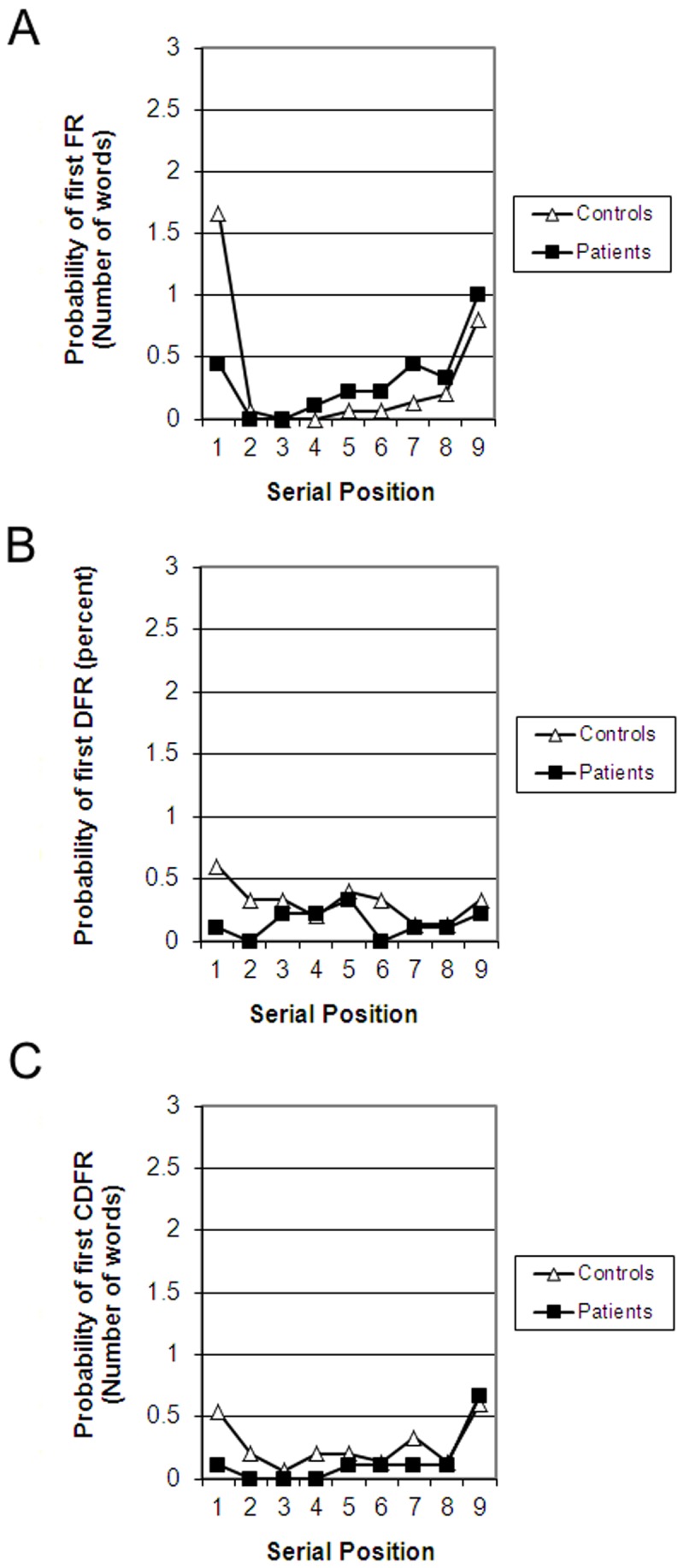
Number of words recalled at the first recall attempt as a function of group and serial position. (a) IFR. (b) DFR. (c) CDFR.

Carlesimo et al. thought that is was important to establish whether patients and controls differed in their retrieval strategy, namely, whether they began their free recall by retrieving early or late list items. To determine whether retrieval strategies differed here, and investigate whether impairments in amnesia are present at the very start of retrieval or unfold over the course of successive retrievals, we analysed the probability of first recall [27, 49, 50]. For each one of the three experimental lists, participants received a score of 1 for each serial position on which they recalled a word and 0 if they recalled nothing or if the first word they recalled was an intrusion, yielding a score of 0–3 for each serial position in each task. Fig 4 shows that patients and controls exhibited an equivalent propensity to recall the most recent list item in IFR (Fig 4a) and CDFR (Fig 4c), but that relative to controls, patients recalled fewer position-1 words in all three tasks.

We analysed the probability of recalling a word from position 1, 5 and 9 with a 3 (task) by 3 (Serial position) repeated-measures ANOVA. There were significant main effects of task [F(2,44) = 4.69, p<.05, partial η^2^ = .18], serial position [F(2,44) = 15.14, p<.001, partial η^2^ = .41], and Group [F(1,22) = 9.32, p<.01, partial η^2^ = .30]. There were significant interactions between Task and Serial Position [F(4,88) = 1.83, p<.05, partial η^2^ = .14] and Group and Task [F(2,44) = 4.69, p<05, partial η^2^ = .17]. To unpack the interaction of Task and Serial Position each task was analysed separately with Serial Position as a within-subject factor and Group as a between-subject factor. There was a significant main effect of Serial Position on IFR [F(2,44) = 6.21, p<.01, partial η^2^ = .22], which exhibited a quadratic trend [F(1,21) = 46.87, p<.001, partial η^2^ = .68]. The main effect of Group was significant [F(1,22) = 6.76, p<.05, partial η^2^ = .64] and Group interacted with Serial Position [F(2,44) = 4.27, p<.05]. Bonferroni-corrected t-tests showed that this interaction was due to the fact that controls recalled more position-1 items [t(22) = 2.93, p<.01] but there was no difference between the groups in recall of position-9 (t<1) or position-5 [t(22) = 1.10, p>.10] items. In CDFR, the only significant effect was that of Serial Position [F(2,44) = 4.13, p<.05], which exhibited a quadratic trend [F(1,22) = 5.25, p<.05]. Because of the importance of the comparison between the groups in recall of position 9, we report this post-hoc comparison despite the absence of a significant interaction [t<1]. In DFR the only significant effect was the main effect of Group [F(1,22) = 4.85, p<.05].

### Free recall of songs

**Fig 5:**
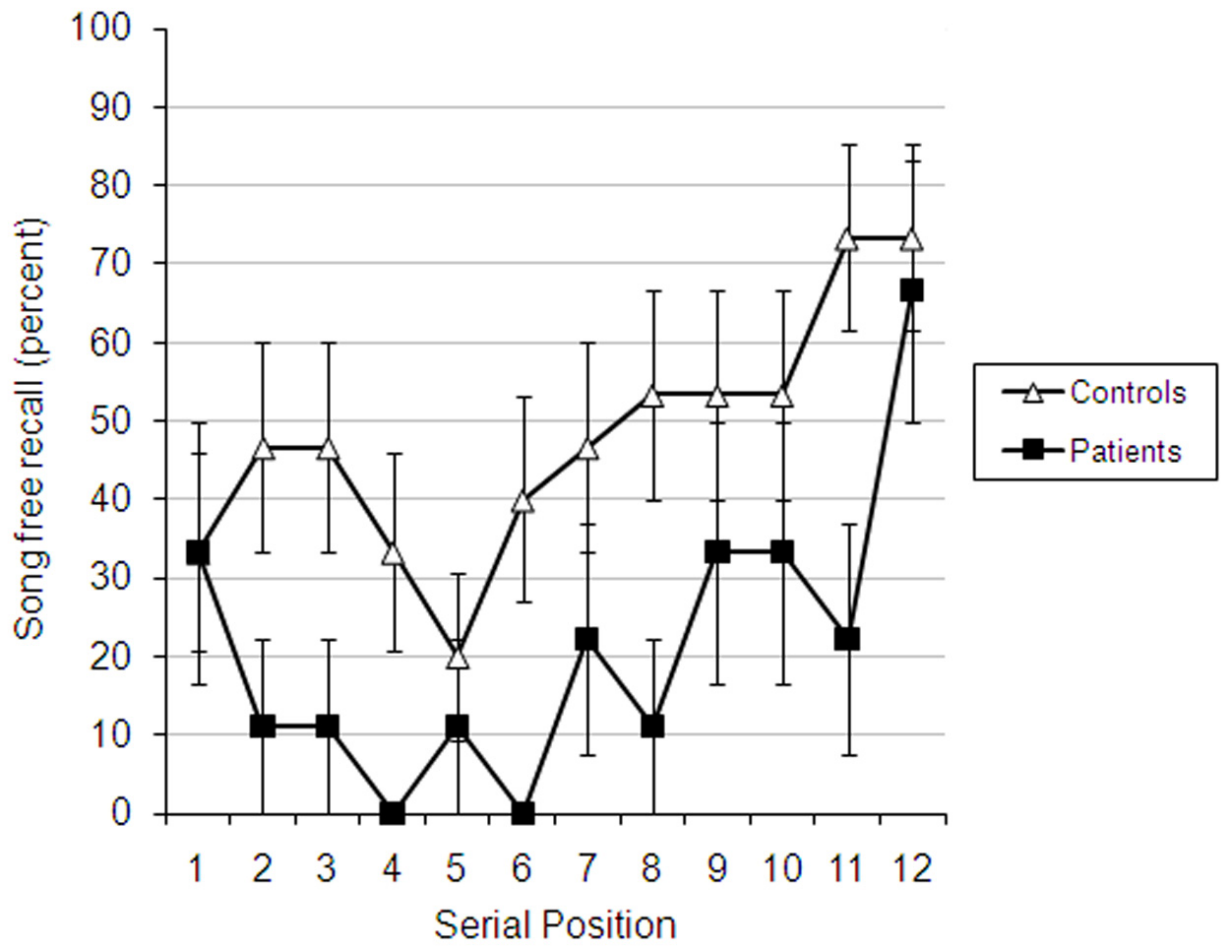
Song recall as a function of group and serial position. Error bars represent standard error.

Every patient was able to recall at least one song (range: 1 to 5 songs, in spite of the fact that some of them voluntarily commented that they did not remember that they had listened to any songs at all (unfortunately, we did not record which patients made such comments). Participants received a score of 1 when they recalled the name of a song (either the real name or the name they had given it immediately after it was presented), and 0 when they did not. Fig 5 shows primacy and recency effects in this task for both patients and controls. Song recall was analysed with a repeated-measures ANOVA, using Group as a between-subject factor. The results replicated the results from CDFR. The main effect of serial position was significant [F(11,242) = 2.74, p<.01, partial η^2^ = .11], and exhibited linear [F(1,22) = 20.11, p<.001, partial η^2^ = .48] and quadratic [F(1,22) = 7.22, p<.05, partial η^2^ = .25] trends consistent with the presence of both recency and primacy effects. Patients recalled fewer songs than controls [F(1,22) = 10.85, p<.01, partial η^2^ = .33] but Group did not interact with Serial Position, F<1.

### Distractor task

The effect of Task (DFR, CDFR) on the number of exercises attempted and on solution accuracy during the distractor task was analysed with two repeated measures ANOVAs with Group as a between-subject factor. The results indicate that patients found the distractor task more difficult than controls in both DFR and CDFR. Patients attempted to solve fewer exercises than controls F(1,22) = 5.44, p<.05, partial η^2^ = .20, and their accuracy was overall lower than that of controls, F(1,22) = 5.21, p<.05, partial η^2^ = .19. All participants attempted more exercises in CDFR than DFR task [F(1,22) = 45.05, p<.001, partial η^2^ = .67; the interaction with Group was non-significant, F(1,22) = 3.68, p = .07]. Accuracy was higher in DFR [F(1,22) = 19.41, p<.001, partial η^2^ = .47].

## Discussion

Our chief finding was an intact long-term recency effect in participants with amnesia, as indicated by their having a memory advantage equivalent to that of controls for recent, as compared to other items. Indeed, patients exhibited intact recency effects in CDFR and in an analogous task, recall of interspersed songs, confirming a key prediction of single-store models. The intact recency effects in the patient group contrasted with their reduced probability of recall across all serial positions, apart from the recency portion of the IFR condition where their free recall was as good as that of controls.

These patient data alone already exhibit all the crucial features of the standard argument against strict dual-store models and in favour of the single-store, interference-theory account. Recency was present in IFR, flattened in DFR, and then returned in CDFR despite the end-of-list distractor being the same in the CDFR and DFR conditions. This already supports single store models, and shows that their argument against dual-store models generalizes to people with amnesia. This is arguably a more direct test than comparisons between groups, because not only are conditions within a group matched on a whole set of variables, but we can also be more certain that a participant’s experience during study was well equated across conditions. The comparison between groups is informative, nonetheless. Specifically, because the full long-term recency pattern was observed in both groups, we can conclude not only that people with amnesia exhibit long-term recency, but that they can do so under conditions in which control participants also exhibit long-term recency.

Our findings qualitatively replicate those of Carlesimo et al. (1996) for the conditions common to both. Comparing their Figure 1 and our Fig 3 suggests that the serial position curve in CDFR is steeper in Carlesimo et al.’s study. This could be due to controls in that study rehearsing the first and last few anagrams throughout the distractor task, because they, unlike patients, could remember that a memory test was coming up. Because we included a DFR condition and used the same procedure across IFR, DFR and CDFR we can be more confident than Carlesimo et al. in comparing across conditions and groups. Had controls used such a strategy in our study they would have also exhibited a recency effect in the DFR condition. Proponents of dual-store models can be reassured, as a consequence of our additional DFR condition, that both groups of participants must have retrieved the recency items in CDFR from long-term memory.

Because the patient groups in the two studies produced quantitatively very similar serial position curves but our controls produced a flatter serial position curve, it may be tempting to attribute the difference between our conclusions and those of Carlesimo et al. to differences between the two control groups. However, clearly, while cross-experiment comparison is typically frowned upon even when experimental conditions are identical, it is not valid here, when the methodologies were different. What is most relevant to our inference is that the full pattern is present in both groups, and that in CDFR the magnitude of the long-term recency effect in patients was similar to that exhibited by their own, matched controls. Crucially, those authors interpreted the reduced levels of free recall in the recency portion of the CDFR task as evidence for a deficient long-term recency effect in amnesia. We were able to show that although patients had poorer memory for words from all serial positions in that task, there was no interaction between serial position and group, suggesting that the long-term recency effect in amnesia was equivalent to that of controls, concluding that this aspect of long-term memory was therefore intact in the patient group.

One potential caveat for the comparison between patients and controls in DFR and CDFR was that patients performed more poorly on the distractor task than controls. This may have influenced the amount of interference the two groups experienced, but from these data we cannot tell whether patients suffered more interference (because they found the distractor task more difficult) or less interference (because they solved fewer arithmetic problems). Notably, the time both patients and controls invested in the distractor task was equivalent.

Clearly, the long-term recency effect in amnesia further discredits classical ‘strict’ dual-store models, suggesting that arguments against these models can be made within the amnesic population as well as in healthy participants. Both single-store and hybrid dual-store models can account for the long-term recency we observed here in both patients and neurologically intact controls, and use interference-based mechanisms to do so. However, this finding has greater theoretical significance for single- than dual- store and hybrid models of memory. Because recency in IFR in amnesia is intact, single-store models are forced to predict intact recency in CDFR in that group as well, namely, predict scale invariance of the recency effect. The intact long-term recency that we demonstrated in amnesia therefore strongly supports these models. By contrast, hybrid dual-store accounts assume memory operates differently in IFR and CDFR, so our finding, while compatible with these models, does not challenge these models, nor does it support them.

Single-store and hybrid dual-store models offer different accounts for the finding that patients’ memory levels only matched that of controls in IFR but not in CDFR. According to hybrid models [4, 18], in IFR, patients utilize their intact STM to recall late list items as effectively as controls. Our analysis of the probability of first recall [25, 26, 30] helps single-store models account for this finding. Strikingly, the qualitative difference in memory between patients and controls across all serial positions in CDFR, apparent when all recall output was considered, was eliminated when analysis was limited to the first recall attempt. We found that at the end of the IFR and CDFR lists, the very last studied item was equally accessible for both patients and controls despite the fact that in CDFR, an effective 30-second distractor interval preceded the cue to begin recall. The impairment patients exhibited in recalling late serial positions in CDFR was not present at the start of recall but emerged later in retrieval. We interpret these results according to Sederberg et al.'s (2008) single-store, interference-theory model, an extension of the Temporal Context Model [27]. It uses a specific representation of context as a retrieval cue, and assumes that when an item is retrieved, its context is retrieved along with it, and that this retrieved context then becomes part of the next retrieval cue. Thus, at the start of free recall, the only cue available is the current context. Current context would be quite similar to the context learned along with list items in IFR, slightly less similar to studied items in CDFR, and least similar to studied list items in DFR (all based on the ratio-rule-like assumption that the distractor task simply acts to accelerate contextual drift). They then propose that amnesia might be understood as impaired learning of new context-item associations, with an intact ability to use current context as a retrieval cue. This kind of mechanism might explain the effects we observed: If cueing with current list context is intact, then this should produce intact retrieval of the most recent items in IFR, and an impairment for all earlier serial positions and for all serial positions in both DFR and CDFR, since those conditions rely more on the use of retrieved context (via item-context associations) as a retrieval cue. However, for the very first recall (corresponding to our probability of first recall measure), current context is the only retrieval route available to controls as well as to patients, so differences should not yet be apparent. For all subsequent output positions, however, retrieved context would be expected to enhance control participants’ ability to retrieve additional items, a mechanism unavailable in amnesia.

The pattern of intact recency in probability of first recall and impaired recency in subsequent recalls is also compatible with the idea that differences in free recall in amnesia might be largely accounted for by output interference effects. Numerous studies have suggested that people with amnesia are more susceptible to various forms of interference than neurologically-intact controls [37, 38, 41], and recent work suggests that cognitive tasks may produce more retroactive interference in amnesia than in controls [39, 40]. It may be that our patients were more distracted by their own responses than controls, and thus, their memory impairment was not evident in the first recall, but was revealed as the recall phase unfolded. This kind of account could be integrated into a broad range of models of free recall, including both single- and dual-store models.

One feature of our data was that people with amnesia had impaired primacy effects (i.e., a reduced advantage in memory for the first few presented items), both when all recall positions were considered and in the analysis of the first recalled position. Brown, Neath & Chater (2007) argued that a reduced primacy effect in IFR in amnesia could be explained as resulting from a failure to rehearse during presentation of the list. As this was not the focus of our study, we do not comment further on this finding.

A caveat in interpreting the results presented here is that people with amnesia recalled very few items in DFR and CDFR. This would not surprise any clinician working with these patients; indeed, clinicians are more likely to be surprised by their intact first recall. However, it is important to bear this floor effect in mind when examining our statistical findings.

We could not obtain proper structural neuroimaging data on many of the patients. For those for whom such data were available, the lesions were not circumscribed to a single structure such as the hippocampus, consistent with the varied aetiologies of their disorder. Our findings, therefore, speak only to the functional consequences of amnesia and not to its structural correlates. The procedure we developed, however, can be used in the future to study a larger sample of patients whose lesions are well documented and determine, thereby, whether the pattern of preserved and deficient performance is associated with any particular lesion or is common across all amnesic syndromes.

### The song task and clinical implications

Despite the fact that the interval between studied items was seconds in the word-CDFR task and minutes in the song-CDFR task, the results were the same in that patients exhibited a recency effect which paralleled that of controls, and recall levels were reduced across all serial positions, including the most recent ones. We also found evidence for primacy effects in the song task, but this was not present in the word list learning task and discussion of it is outside the scope of our paper. The patients recalled 44% of the two most recently studied songs (serial positions 11–12), more than twice the number of songs they had recalled from midlist positions (11% from serial positions 6 and 7). This finding was obtained despite the fact that between the very last song and song recall, they performed the digit span task, and the second-to-last song ended at least 140 seconds before the very last song. Patients have also exhibited facilitated recall of the first song they heard, more than an hour before they were asked to recall it. Finally, every patient was able to recall freely at least one song. This observation suggests that under some conditions free recall may not reflect recollection as is traditionally assumed [51, 52], but explicit retrieval of primed or ‘activated’ traces, a possibility that has been suggested by some investigators [4]. Rehabilitation scientists could build on these findings to help patients in their care. One straightforward way to implement this finding in aid of rehabilitation is to consider serial position effects when patients are exposed to a new environment. For example, in the Memory Link program (http://research.baycrest.org/mlp) patients are taught to use hand-held devices to record their daily experience. Our findings suggest that the patients may be more likely to remember the last items in a spaced learning situation, so that when given instructions about the use of such devices, one can improve training by spacing out the sessions and prioritize the most important material by placing it at the end of the training. Although memory for one or two items, such as the songs used here, may not seem like much to neurologically intact readers, it can be precious to people with dense amnesia. For example, one of the patients was gratified that he could remember that he heard an excerpt from “Somewhere over the Rainbow”, commenting that this is his daughter’s favourite song.

## Conclusion

People with amnesia clearly have some preserved ability to retrieve items from the long-term store, as Warrington and Weiskrantz (1968) argued when they found that while the patients were recalling the current list, there were intrusions from previous lists. This finding is important because it suggests avenues to pursue in rehabilitation. Our finding suggests that this preserved ability likely obeys the rules described by single-store interference-based models, such as the ratio rule, and confirms a key prediction of single-store models or memory, although it does not contradict hybrid ‘modern’ dual-store models. The finding of intact recency and long-term recency for the first item recalled on each list provides an important constraint on possible accounts of the memory deficit in amnesia.
